# Comparative skin microbiome analyses reveal differences between wild populations and captive groups of the Montseny brook newt (*Calotriton arnoldi*)

**DOI:** 10.1093/ismeco/ycaf245

**Published:** 2026-01-08

**Authors:** Sergi Tulloch, Maria Estarellas, Dean C Adams, Anthony Bonacolta, Viviana Pagone, Daniel Fernández-Guiberteau, Fèlix Amat, Albert Montori, Francesc Carbonell, Elena Obon, Mónica Alonso, Marta Santmartín, Josep Xarles, Rosa Marsol, Daniel Guinart, Sònia Solórzano, Adrián Talavera, Bernat Burriel-Carranza, Elena Bosch, Javier del Campo, Salvador Carranza

**Affiliations:** Institut de Biologia Evolutiva (CSIC-Universitat Pompeu Fabra), Pg. Marítim de la Barceloneta 37-49, Barcelona 08003, Catalonia, Spain; Institut de Biologia Evolutiva (CSIC-Universitat Pompeu Fabra), Pg. Marítim de la Barceloneta 37-49, Barcelona 08003, Catalonia, Spain; Department of Ecology, Evolution and Organismal Biology, Iowa State University, Ames, Iowa 50011, United States; Institut de Biologia Evolutiva (CSIC-Universitat Pompeu Fabra), Pg. Marítim de la Barceloneta 37-49, Barcelona 08003, Catalonia, Spain; Department of Marine Biology and Ecology, Rosenstiel School of Marine Atmospheric and Earth Science, University of Miami, Miami, Florida, 33149-1031, United States; Institut de Biologia Evolutiva (CSIC-Universitat Pompeu Fabra), Pg. Marítim de la Barceloneta 37-49, Barcelona 08003, Catalonia, Spain; CREAC - Center de Recerca i Educació Ambiental de Calafell (GRENP-Ajuntament de Calafell), Calafell 43882, Catalonia, Spain; Àrea d’Herpetologia, BiBIO, Museu de Granollers – Ciències Naturals. Palaudàries 102, Granollers 08402, Catalonia, Spain; CREAC - Center de Recerca i Educació Ambiental de Calafell (GRENP-Ajuntament de Calafell), Calafell 43882, Catalonia, Spain; Àrea de Gestió Ambiental Servei de Fauna i Flora (Center de Fauna de Torreferrussa), Santa Perpètua de Mogoda 08130, Catalonia, Spain; Àrea de Gestió Ambiental Servei de Fauna i Flora (Center de Fauna de Torreferrussa), Santa Perpètua de Mogoda 08130, Catalonia, Spain; Àrea de Gestió Ambiental Servei de Fauna i Flora (Center de Fauna de Torreferrussa), Santa Perpètua de Mogoda 08130, Catalonia, Spain; Zoo de Barcelona, Barcelona 08003, Catalonia, Spain; Zoo de Barcelona, Barcelona 08003, Catalonia, Spain; Àrea de Gestió Ambiental Servei de Fauna i Flora (Center de Fauna del Pont de Suert), El Pont de Suert 25520, Catalonia, Spain; Servei de Gestió de Parcs Naturals, Diputació de Barcelona, Barcelona 08008, Catalonia, Spain; Servei de Gestió de Parcs Naturals, Diputació de Barcelona, Barcelona 08008, Catalonia, Spain; Institut de Biologia Evolutiva (CSIC-Universitat Pompeu Fabra), Pg. Marítim de la Barceloneta 37-49, Barcelona 08003, Catalonia, Spain; Institut de Biologia Evolutiva (CSIC-Universitat Pompeu Fabra), Pg. Marítim de la Barceloneta 37-49, Barcelona 08003, Catalonia, Spain; Museu de Ciències Naturals de Barcelona, P° Picasso s/n, Parc Ciutadella, Barcelona 08003, Catalonia, Spain; Institut de Biologia Evolutiva (CSIC-Universitat Pompeu Fabra), Pg. Marítim de la Barceloneta 37-49, Barcelona 08003, Catalonia, Spain; Department of Medicine and Life Sciences, Institute of Evolutionary Biology (UPF-CSIC), Barcelona 08003, Catalonia, Spain; Institut de Biologia Evolutiva (CSIC-Universitat Pompeu Fabra), Pg. Marítim de la Barceloneta 37-49, Barcelona 08003, Catalonia, Spain; Institut de Biologia Evolutiva (CSIC-Universitat Pompeu Fabra), Pg. Marítim de la Barceloneta 37-49, Barcelona 08003, Catalonia, Spain

**Keywords:** microbiome, captivity, amphibians, conservation, reintroduction, newts

## Abstract

The Montseny brook newt, *Calotriton arnoldi*, is a Critically Endangered amphibian species endemic to the Montseny Massif in Catalonia, Northeastern Spain. Due to population declines and threats to its natural habitat, an *ex-situ* breeding program was initiated in 2007. A key goal of the program is to ensure the survival of captive-bred individuals after reintroduction, which in amphibians heavily relies on the specimens’ microbiome being capable of protecting them from environmental microorganisms, especially considering the global Chytridiomycosis pandemic caused by the fungi *Batrachochytrium dendrobatidis* (*Bd*) and *Batrachochytrium salamandrivorans* (*Bsal*). This study aims to characterize the skin microbiome of wild and captive *C. arnoldi* specimens and identify differences in their composition, contributing to future research on the microbiome’s impact in captive-bred individuals upon reintroduction. Up to 5996 ASVs (Amplicon Sequence Variants) were identified from 138 samples from 21 and 61 wild and captive-bred individuals, respectively. Results indicate that wild populations from different subspecies have significantly different skin microbiome composition, as do wild and captive-bred groups from the same subspecies.

Additionally, dissimilarities in skin microbiome variability were only found within each subspecies, between wild and captive-bred groups. In terms of composition, certain bacteria were identified as potential markers for both wild and captive environments. Enhancing skin microbiome variability might improve the survival prospects of reintroduced specimens. Thus, exposing captive specimens to a more natural environment while in captivity or a soft-release procedure could potentially mitigate the absence of exposure to other bacteria and potential pathogens from their native environment.

## Introduction

Recent studies based on the analysis of 32% of terrestrial vertebrate species indicate that, beyond the ongoing global extinctions, our planet is undergoing a rapid decline and disappearance of natural populations, referred to as “biological annihilation” [1]. Over the past decades, factors such as overexploitation, habitat loss, the introduction of invasive species, pollution, climate change, and emerging diseases have led to a catastrophic decline in the number and size of vertebrate species populations [2, 3]. As a result, in the last 100 years, hundreds of species and vertebrate populations have become extinct at a rate 100 times higher than the natural extinction rate over the past two million years, suggesting we are already in the sixth major episode of mass extinction on our planet [1, 3].

Among all groups of terrestrial vertebrates, amphibians have received significant attention in the last four decades. Despite having survived several mass extinctions, evidence indicates that more amphibian species have already gone extinct or are endangered compared to other vertebrate groups [3]. A reason for such increased susceptibility is their permeable skin, allowing the absorption of water and gases for respiration and hydration, but, at the same time, making them especially susceptible to pollution, climate change-related events (such as changes in temperature or precipitation patterns), and emerging diseases.

One of the most significant challenges amphibians face is the chytridiomycosis pandemic, caused by the chytrid fungi *Batrachochytrium dendrobatidis* (*Bd*) and *Batrachochytrium salamandrivorans* (*Bsal*), already leading to the disappearance of over 200 amphibian species worldwide, with many more predicted to become extinct in the near future [4–5]. Chytrids are fungi that usually live in soil or water but occasionally parasitize other fungi, plants, or insects. Importantly, *Bd* and *Bsal* are the only known chytrids that infect vertebrates. These species remain as spores in the water until they encounter a host. At this point, they encyst, fructify, and proliferate throughout the host’s keratinized body parts (i.e. mouthparts in larval stages and skin in adults) [6–7], disrupting the normal regulatory functioning of the amphibians’ skin [8].

Because of its permeability, the amphibian skin has a very important microbial component based on bacteria, fungi, and protists; yet, how amphibians obtain their microbiota remains unclear. Evidence suggests that individuals may acquire their microbiome from the environment, both through horizontal transmission (e.g. during mating or communal gatherings) [9] and vertical transmission (particularly in species that exhibit parental care) [10]. The microbiome has a major influence on many processes, including the host’s digestion, behavior, development, and reproduction [11–13], but what is of most interest for this study is the pivotal role it has as an integral part of the immune system [14]. Harris *et al*. [15] demonstrated that some skin bacteria inhibit the growth of chytrid fungi, emphasizing the importance of the individual’s microbial community and its impact on disease survival. Moreover, it has been shown that some amphibians can enhance their chances of survival against chytrid fungi if they have been previously exposed to a milder strain of the fungus [16].

The Montseny brook newt, *Calotriton arnoldi*, is a species endemic to the Montseny Massif in eastern Catalonia, formally described by Carranza and Amat in 2005 [17]. Its area of occupancy is limited to eight streams in less than 10 km^2^, making it the most threatened amphibian species in Europe and being considered Critically Endangered by the IUCN [18]. Bearing in mind that *C. arnoldi* is a completely aquatic urodele at both larval and adult stages, it faces constant threats, including water overexploitation, deforestation, stream continuity disruption, warming temperatures, natural disasters, and emerging diseases like chytridiomycosis. This is particularly concerning given that a recent *Bd* and *Bsal* outbreak was detected in the Montnegre i el Corredor Natural Park, located just 15 km south of the natural range of *C. arnoldi,* and even more so given that *Bsal* is lethal to this species [19].

Recently, two subspecies of *C. arnoldi* have been recognized: *C. a. laietanus*, comprising five populations located to the west of the Tordera River (Western populations: B1–B5), and *C. a. arnoldi*, consisting of three populations to the east (Eastern populations: A1–A3) [17, 20]. The census of the species is also worryingly low, indicating that only 1000–1500 individuals remain in their natural habitats [17]. In response to the critical conservation status of the species, an *ex-situ* breeding program was initiated in 2007, followed by the launch of a LIFE project in 2016 (LIFE15 NAT/ES/000757) aimed at improving the chances of the species’ survival.

Although *ex-situ* breeding programs are valuable tools for species recovery, they also present certain drawbacks. These include potential risks, such as adaptation to captivity—where traits favored in captive conditions may reduce fitness in the wild—loss of genetic diversity or changes in microbial communities between wild and captive populations, which are increasingly recognized as critical to host immunity and environmental resilience [21–23]. These concerns are particularly relevant in this case, as some genetic clusters from different geographic locations are not currently represented in the breeding program [24], which not only limits the genetic diversity captured *ex-situ* but may also result in population-specific microbiomes being overlooked.

These findings raise important questions about how captivity may be affecting the microbiome of *C. arnoldi*, whether wild populations differ in their microbial communities, and what the implications of these differences might be for reintroduction success. To address these questions, this study characterizes the skin microbiome of *C. arnoldi* by collecting swabs from wild and captive individuals of both subspecies to test the hypotheses that (A) wild populations of different subspecies will exhibit distinct microbiomes due to genetic [25] and environmental divergence [26–28] and (B) captive populations will show altered or less diverse microbiomes relative to their wild counterparts [29–31], potentially impacting their survival upon reintroduction. The generated data are also used to assess the microbiome’s potential role in species survival under both captive conditions and after reintroduction.

## Materials and methods

### Study system and sampling

To explore these questions and test our hypotheses, we designed a sampling strategy incorporating individuals from both wild and captive populations, across subspecies and generations, and spanning the three breeding facilities currently involved in the conservation program.

The initial breeding center, the Torreferrussa Wildlife Recovery Center, housed founding individuals from both subspecies: wild Western *C. a. laietanus* (B1 and B2 populations) and wild Eastern *C. a. arnoldi* (A1 population). These subspecies were kept in separate enclosures, reflecting their natural geographic separation. Six years later, the Barcelona Zoo joined the program. Founding individuals at this center consisted exclusively of first-generation (F1) newts bred at Torreferrussa for both subspecies. In 2013, the breeding program expanded to include the Pont de Suert Wildlife Recovery Center, which also began with first-generation *C. a. laietanus* individuals from Torreferrussa.

At all facilities, adult newts were housed in water tanks in groups of four (two males and two females), while juveniles were housed together according to their subspecies. Water in the tanks consisted of a mixture of reverse osmosis water and tap water (1:9 ratio), left to stand for 24–48 hours. No biological organisms were added beyond frozen and live food (e.g. mosquito larvae, *Gammarus*, *Daphnia*, *Artemia*, *Tubifex*, *Tribolium…*). Water management varied slightly among centers: in Torreferrussa and Pont de Suert, one-third of the tank volume was replaced every three weeks; while in Barcelona Zoo water exchange was determined based on water quality analyses, with small daily replacements and a larger change every two months.

Based on this setup, skin microbiome samples were collected from both subspecies of wild populations and all available breeding centers, ensuring separation of individuals during sampling to avoid pseudo-replication. Following Bletz *et al*. [32], two skin swabs were taken per individual to enhance microbial yield, and individuals were sexed during sampling whenever possible to minimize sex-based bias.

**Figure 1:**
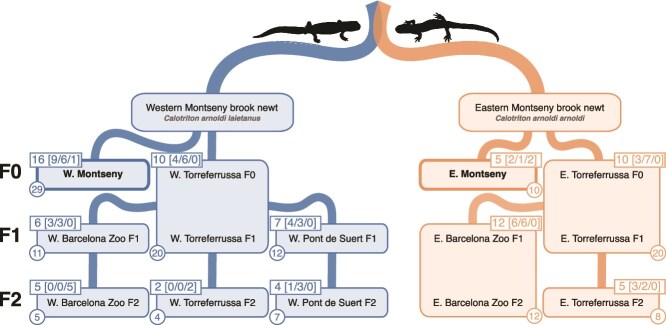
Summary of sample sorting, including subspecies, location, generation, assigned group, number of sampled specimens and their sexes, and amount of skin microbiome samples per group. All group names were simplified according to their subspecies (W, Western Montseny brook newt [*C. a. Laietanus*]; E, Eastern Montseny brook newt [*C. a. Arnoldi*]), location, and the generations they contain (F0, F1, and/or F2). Bold frames and names indicate wild samples. Numbers in the upper corner rectangles indicate sampled specimens as following: Total number (females/males/juveniles), and numbers in the lower corner circles indicate samples of skin microbiome from the group. Silhouettes were extracted from images by a. Talavera.

A total of 138 microbiome skin samples of *C. arnoldi* were collected from 82 *C. arnoldi* individuals during May 2022 and between March and April 2023, spanning all subspecies, all centers and generations from F0 to F2 (Fig. 1).

For each group of *C. arnoldi*, a water swab sample was collected from river (wild populations) or from the housing water (captive populations). Groups were considered isolated when maintained in completely separate water systems. Based on this criterion, all individuals were classified into eleven distinct groups (Fig. 1), which serve as the basis for most of the statistical analyses conducted in this study.

Sampling was conducted according to the sanitary protocol of the Generalitat de Catalunya [33] to avoid contamination and the spread of emerging diseases. Permits to carry out this work were granted by the Wildlife Service of the Generalitat de Catalunya and the Area of Natural Parks of the Diputació de Barcelona.

### DNA extraction from swabs and sequencing

DNA was extracted from swabs using the DNeasy Blood and Tissue Extraction kit (QIAGEN), although samples were submitted to a prior lysis step involving lysis buffer (10 mL of Tris-Cl 100 mM, 1 mL of 100 mM sodium EDTA, 600 μL of Triton X-100 and 38.4 mL of distilled water), lysozyme (20 mg per mL) and a short incubation (1 hour at 37°C) [32]. The V4 region of the bacterial 16S ribosomal RNA gene was amplified with a PCR following an adapted protocol developed by the Earth Microbiome Project [34–37]. The PCR reaction per sample included 0.5 μL of both forward (515 F: 5′-TCG TCG GCA GCG TCA GAT GTG TAT AAG AGA CAG GTG YCA GCM GCC GCG GTA A-3′) and reverse (806 R: 5′-GTC TCG TGG GCT CGG AGA TGT GTA TAA GAG ACA GGG ACT ACN VGG GTW TCT AAT-3′) primer and adapter (10 μM;), 22.5 μL of Taq SuperMix (Invitrogen Platinum PCR SuperMix, High Fidelity) and 3 μL of the extracted DNA. PCR conditions were as follows: denaturalization at 94°C for 3 minutes, followed by 30 cycles of 30 seconds at 94°C, 30 seconds at 52°C, and 30 seconds at 68°C, with a final extension step at 68°C for 4 minutes. Subsequently, PCR products were visualized in a 1% agarose gel. Finally, PCR products were sequenced for an average of 50 000 reads per sample using paired-end 2x250 v2 chemistry on Illumina MiSeq at the Genomics Core Facility of the Pompeu Fabra University (Barcelona Biomedical Research Park, Barcelona, Spain). The DNA gene amplicon reads will be deposited in the NCBI Sequence Read Archive (PRJNA1265703).

### Bioinformatic processing

All statistical analyses were performed in R v.4.3.1 [38]. Each read was trimmed of its primers and sequencing adapters using Cutadapt [39]. Then, DADA2 v.1.28.0 [40] was used to assess read errors, truncate reads, and merge paired-end reads, followed by chimera removal. Afterwards, Amplicon Sequence Variants (ASVs) were inferred using a Bayesian classifier with the Silva v. 138.1 database [41]. The Phyloseq v.1.44.0 package [42] was used to manipulate the amplicon sequence data within R, as well as to assess alpha diversity and generate relative abundance plots. Lab/kit contaminants were removed from the ASV table using the R package DECONTAM v.1.20.0 [43] and PCR negative control samples. Chloroplast, mitochondrial, eukaryotic, and embryophyte ASVs were also removed, as were the environmental control samples. Low-prevalence ASVs were filtered out by keeping only ASVs with two reads in at least two specimens. Following this quality control step, the final dataset comprised 7 420 474 processed reads assigned to 5996 unique ASVs (Table S1). In total, 138 skin microbiome samples from 82 *C. arnoldi* individuals were retained (Fig. 1), along with 11 water samples, which were kept for descriptive comparisons of the microbiomes but removed for statistical analyses.

### Microbiome analysis

To account for the compositional nature of the data, a center-log-ratio transformation of the final dataset was performed using the microbiome package v.1.28.0 [44]. For individuals with multiple samples, read counts were summed before calculating relative abundances. Microbiome transferability across generations was assessed as changes in average ASV relative abundances, with standard deviations calculated for each group. Ampvis2 v.2.8 [45] was then used to generate heatmaps and identify core ASVs within each group, which were defined as those shared by at least 75% of the individuals with a relative abundance ≥0.1%.

The final dataset (Table S1) was divided into three subsets for downstream analyses: (i) Wild individuals from both subspecies, to test Hypothesis A; (ii) all *C. a. laietanus* (wild and captive), to test Hypothesis B within the Western subspecies, and (iii) all *C. a. arnoldi* (wild and captive), also to test Hypothesis B but with the Eastern subspecies.

Alpha diversity was assessed with the Shannon Diversity Index [46]. Wilcoxon rank-sum tests, adjusting *P*-values with the Bonferroni correction, were used to compare alpha diversity between the two subspecies (Dataset 1) and between wild populations and each group of captive individuals within subspecies.

Beta-diversity was addressed using the Aitchison distance. To test for group-level differences in community composition, a PERMANOVA was performed using the RRPP v.1.3.1 package [47] (with 10 000 permutations), testing in each dataset for the effect of *Groups* (Fig. 1). The *Centers* variable was not included in the model to avoid collinearity, as it was highly associated to *Groups* (χ^2^ = 246, df = 30, *P* < 2.2·10^−16^). Where the overall PERMANOVA indicated significant differences, *post-hoc* pairwise comparisons between groups were conducted using the *pairwise()* function in RRPP with Bonferroni corrections; and pairwise results were summarized for both centroid differences (*test.type = “dist”*) and group-level dispersion (*test.type = “var”*).

ANCOMBC v.2.2.0 [48] was used to determine significantly different relatively abundant ASVs (when [|Log_2_ (FC)|] > 2 and *P*-value <.05) between subspecies in the Wild dataset (i) to address hypothesis A; and in the Western (ii), and Eastern (iii) datasets independently to address hypothesis B. BLAST [49] was used to identify relevant ASVs when the Silva database taxonomy was not sufficiently specific.

## Results

### Compositional and transferability analysis

**Figure 2:**
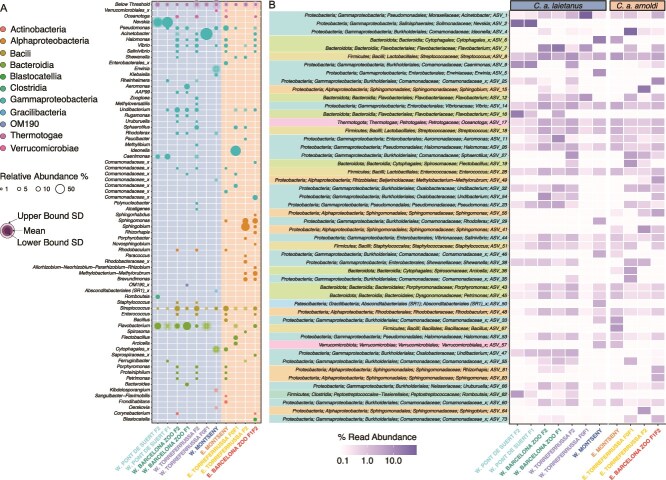
Microbiome composition of wild populations and captive-bred groups of *C. Arnoldi*. (A) Bubble plot representing genera with >1% relative abundance in at least one of the wild populations and captive-bred groups and (B) heatmap representing the most relative abundant ASVs among the newt groups; both color-coded according to taxonomic class. W, Western Montseny brook newt (*C. a. Laietanus*); E, Eastern Montseny brook newt (*C. a. Arnoldi*).

*Calotriton arnoldi*’s microbiome mainly comprises two phyla: Proteobacteria and Bacteroidota. Most of the Proteobacteria belong to Gammaproteobacteria, including the most relatively abundant genera, which can dominate an entire group, like *Acinetobacter* in W. Torreferrussa F0F1 or *Nevskia* sp. in both Pont de Suert F1 and F2 groups (Fig. 2A and B). Interestingly, all groups share *Streptococcus* ASVs, and most groups also have *Pseudomonas* spp. Some genera seem to be characteristic of wild microbiomes, having higher abundances in the wild populations and minimal in captive ones, such as an unclassified species from the genera *Cytophagales* or *Verrucomicrobiales.* On the other hand, captive-bred groups share several ASVs, including *Flavobacterium*, specifically ASV 7, 12, and 16 (Fig. 2B). Despite close clustering of samples from captive-bred individuals to water samples in the PCA, water samples had fewer ASVs with different composition than newt microbiomes (Fig. S1 and Table  S2). For example, water samples from all breeding centers were dominated by ASV 8, a *Streptococcus,* which was also detected in the wild water but at much lower relative abundance.

**Figure 3:**
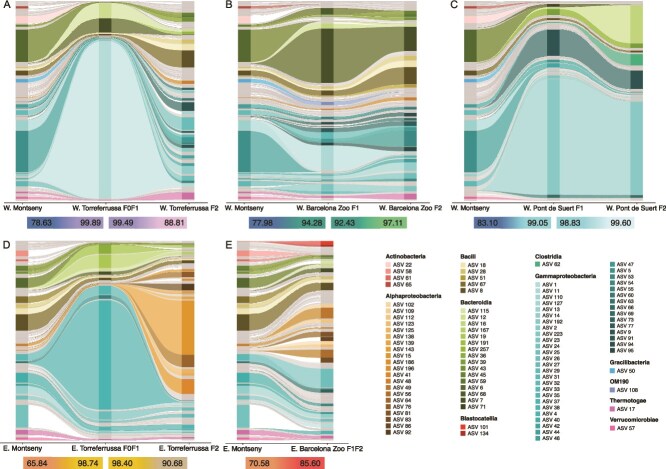
Microbiome transferability and mean relative abundance of shared ASVs across breeding centers of both subspecies of *C. Arnoldi.* ASVs are colored according to taxonomic class when their relative abundance equals or exceeds 1%. The percentages of total shared ASVs between groups are shown below each graph. W, Western Montseny brook newt (*C. a. Laietanus*); E, Eastern Montseny brook newt (*C. a. Arnoldi*).

Microbiome transferability from wild populations to captive F0, F1, and F2 generations was evaluated by comparing the average relative abundance of shared ASVs between wild and captive groups (Fig. 3; Fig. S3). From this it was revealed that the wild populations of *C. arnoldi* shared over 90% of their microbiome (Fig. S2), a higher proportion than any of the captive-breeding groups with their respective wild group. In both the Barcelona Zoo and Pont de Suert breeding centers, the microbiome tended to remain stable across generations (Fig. 3). However, they still lacked a major percentage of bacteria present in wild newts (such as ASV 5 and 6) and had notable differences in their relative bacteria abundances. As for the Torreferrussa breeding center, the founders and first-generation groups (W. & E. Torreferrussa F0F1) seem to have gone through a microbiome bottleneck, leaving few ASVs to dominate their microbiome. In contrast, F2 generations from this center (W. & E. Torreferrussa F2) seem to be improving their microbiome’s repertoire (Fig. 3). Moreover, F2 groups from both subspecies seem to diverge in their microbiome compared to the wild populations (Fig. S2). Notably, the proliferation of Alphaproteobacteria in the *C. a. arnoldi* F2 groups was remarkable (Fig. 3D and E), as they do not have much presence in the wild group nor in E. Torreferrussa F0F1 group. Yet, they considerably expand in the E. Torreferrussa F2 group and in the Barcelona Zoo F1F2 group.

### Core microbiome

**Figure 4:**
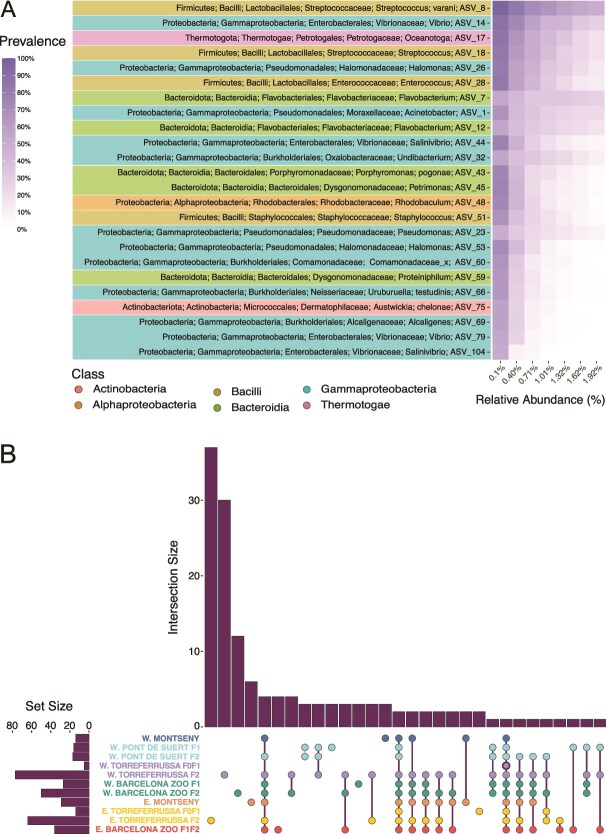
Core bacteria of *C. arnoldi’*s microbiome. (A) Heatmap representing core ASVs for all samples pooled together, colored according to taxonomic class. (B) UpSet diagram for shared core ASVs across wild populations and captive-bred groups. W, Western Montseny brook newt (*C. a. Laietanus*); E, Eastern Montseny brook newt (*C. a. Arnoldi*).

When analysing the full set of *C. arnoldi* samples together, the species-level core microbiome comprised 9 ASVs, with ASV 8 standing out by being both relatively abundant and consistently present across the dataset (Fig. 4A). Examining the core microbiome within each wild population and captive-bred group, ASV 8 was also consistently detected as a core ASV across all populations and groups (Fig. 4B). ASVs 14 (*Vibrio* sp.) and 17 (*Oceanotoga* sp.) were core bacteria in all groups except the W. Torreferrussa F0F1 group, which was dominated by ASV 1 at 78.94% relative abundance (Table S2). Additionally, seventeen core ASVs were identified across the wild populations altogether, but only eleven of them were shared core bacteria between the two populations (Table S3). Among captive-bred groups, no core ASVs were shared across all groups except for ASV 8. However, *Undibacterium* sp. (ASV 47) appeared as a distinguishing feature in *C. a. laietanus* captive-bred groups (excluding W. Torreferrussa F0F1), while a different *Undibacterium* sp. (ASV 32) and *Sphaerotilus* sp. (ASV 27) were found to characterize the *C. a. arnoldi* captive-bred groups (Table S3).

### Diversity analysis

**Figure 5:**
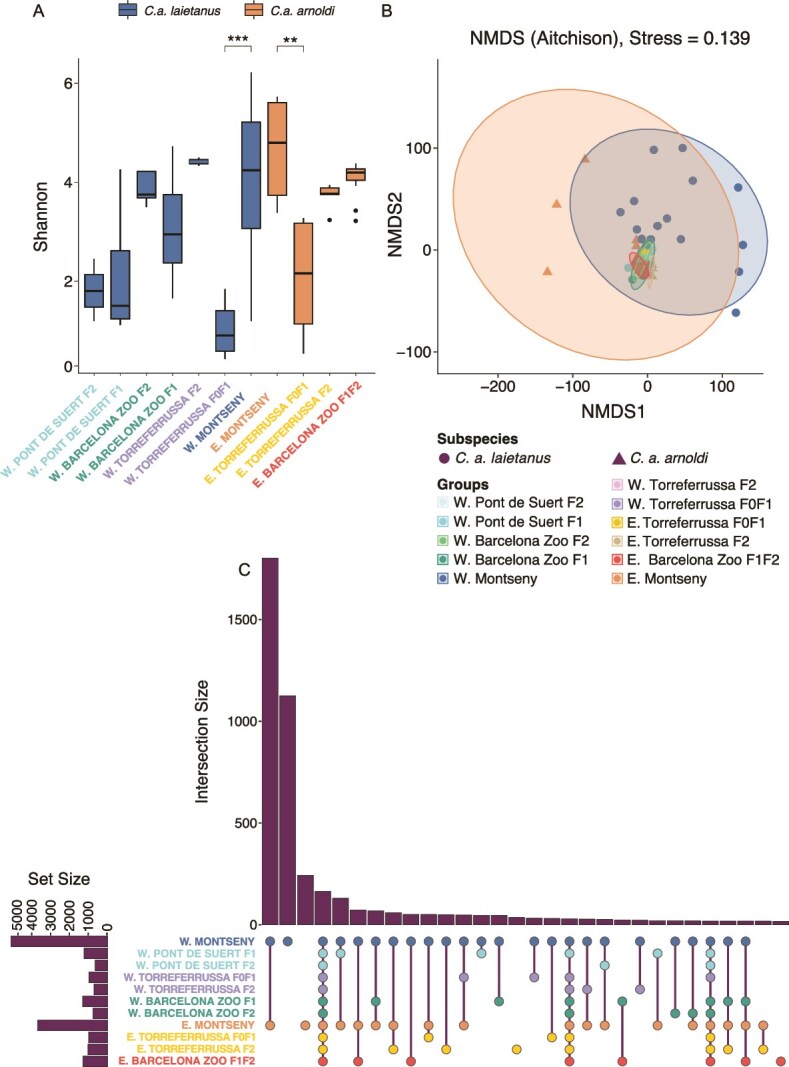
Alpha diversity, diversity through compositional and variance differences, and shared ASVs across wild populations and captive groups of *C. Arnoldi.* (A) Wild and captive-bred comparison of Shannon’s diversity index, with *P*-value for pairwise comparison indicated above (0.05 “^*^” 0.01 “^**^” 0.001 “^***^” 0). (B) Aitchison distance NMDS with 95% confidence interval ellipse for wild and captive-bred groups separated by subspecies. Stress = 0.139. (C) UpSet diagram for shared ASVs across all groups of *C. Arnoldi.* W, Western Montseny brook newt (*C. a. Laietanus*); E, Eastern Montseny brook newt (*C. a. Arnoldi*).

To approach our first hypothesis (A), we compared the microbiomes of the two *C. arnoldi* subspecies using only wild individuals (dataset 1). The Shannon Diversity Index did not differ between subspecies (W = 31, *P* = .49, Fig. 5A). However, according to the PERMANOVA, microbiome composition differed significantly between the two subspecies (*R*^2^ = 0.114, F = 2.44, *P* = .0015), yet beta diversity did not differ in dispersion stats between the two wild populations (RRPP pairwise test for variance: Z = 0.03, *P* = .50).

Likewise, within each subspecies, we then compared the captive-bred groups to the wild populations to tackle our second hypothesis (B). In the *C. a. laietanus* dataset, the Shannon Diversity Index only displayed significant differences after Bonferroni correction between the wild population and the W. Torreferrussa F0F1 group (W = 6; *P* < .0001, Fig. 5A). Regarding PERMANOVA, the *Groups* variable had a significant effect on microbiome composition (*P* < .0001, Table S4), and *post-hoc* comparisons indicated that microbiome composition and beta diversity did not differ between groups from the same center but did differ when compared to their wild counterpart (Table S4), except for the W. Torreferrussa F2 group However, its small sample size (*n* = 2, Fig. 1) may limit statistical power and affect the reliability of this results (Fig. 5B).

For *C. a. arnoldi*, results were very similar to those of *C. a. laietanus*. The Shannon Diversity Index only showed significant differences between the wild population and the E. Torreferrussa F0F1 group (W = 0; *P* = .002, Fig. 5A). PERMANOVA also indicated that *Groups* had a significant effect (*P* < .0001, Table S4) and *post-hoc* comparisons highlighted the same pattern as in the Western subspecies, were microbiome composition and beta diversity did not differ between groups from the same center but did differ when compared to their wild counterpart (Table S4), except for the E. Torreferrusa F2 groups’ composition.

This pattern is evident in the NMDS ordination analyses based on Aitchison distances (Fig. 5B). Reinforcing this idea, the wild populations were found to share more ASVs between their microbiomes than captive*-*bred groups presented in their whole microbiome (Fig. 5C).

### Differential relative abundance

To further explore microbiome differences, ANCOM-BC analyses were conducted on selected pairwise comparisons according to our hypotheses: between wild populations of each subspecies (dataset 1) to address hypothesis A and wild populations versus each captive-bred group for each subspecies (datasets 2 and 3) to assess hypothesis B (Table S5). None of the core ASVs identified in the wild populations showed significant differential relative abundance when comparing the two subspecies. In fact, only 31 ASVs were significantly more relatively abundant in the wild Western Montseny brook newt (*C. a. laietanus*). In contrast, 9 ASVs were significantly more relatively abundant in the wild Eastern Montseny brook newt (*C. a. arnoldi*). Interestingly, the same ASVs tended to be significantly more relatively abundant in the wild populations compared to the captive-bred groups of the same subspecies when compared one by one (Fig. S4). These were all core ASVs unique to the wild populations (i.e. ASVs 6, 33, and 50) or even only to the *C. a. laietanus* population (i.e. ASVs 29, 46, and 57), making it more striking for this pattern to appear in the *C. a. arnoldi* subspecies.

Apart from the W. Torreferrussa F0F1 and F2 groups, few significant differences were detected between captive-bred groups originating from the same center (Fig. S4).

## Discussion

This study described the microbiome of *C. arnoldi*, a Critically Endangered newt with a breeding program that aims to reintroduce captive-bred specimens into their natural habitat.

Results show that *C. arnoldi* subspecies had significantly different microbiomes but had similar variance within each one. These differences could be due to their habitat, as *C. a. laietanus* tends to live in warmer streams covered by holm oaks, whereas *C. a. arnoldi* inhabits streams covered by beech and other deciduous trees [20]. This could mean that each subspecies has acclimatized its skin microbiome to suit its environment better, as seen in previous studies [26]. Nonetheless, both subspecies share certain core bacterial taxa, such as *Streptococcus varani*, *Oceanotoga* sp*.*, or *Vibrio* sp., suggesting that they may be a part of the natural state of this species and that they might have an impact on the survivability of the Montseny Brook Newt. However, similar interpopulation patterns in skin microbiomes have also been documented in wild amphibians, where variation is frequently attributed to environmental or geographic range differences rather than to host-related factors [27, 28, 50, 51, but see 25].

Captive-bred groups showed lower alpha diversity values than their wild counterparts and differed in microbiome composition. This is not unexpected, as the amphibian microbiome changes depending on sex [52], diet [53], habitat [54, 55], season [56, 57] or under captivity [29, 30]. In fact, this has previously been found in red-backed salamanders (*Plethodon cinereus*), where captivity and husbandry conditions altered their skin microbiota by limiting access to natural bacterial reservoirs, such as soil and untreated water, leading to depauperate and atypical communities [31]. However, since captive specimens are under much more stable and controlled conditions, it was surprising that the Torreferrussa F0F1 and F2 groups of both subspecies were so different from one another (Fig. 3). This is especially surprising given that they are under very similar environmental conditions with limited opportunities for microbial acquisition, and more so considering amphibians have been associated with vertical transmission of bacteria [10]. In this context, the three most relatively abundant ASVs in the W. Pont de Suert F2 group were entirely absent from our water sample, suggesting that environmental acquisition alone is unlikely. While it is possible that some taxa survive better on amphibian hosts than in water, the high abundance of these ASVs on the specimens’ skin indicates a potential role for vertical transmission. Overall, the marked difference in bacterial relative abundances between wild and captive-bred samples is a striking finding. Such discrepancies may have important implications for the survival of reintroduced *ex-situ* individuals, as the missing bacterial taxa could play a key role in conferring resistance to natural pathogens, including the chytrid fungi responsible for chytridiomycosis. Further research is needed to assess the functional role of *C. arnoldi*’s microbiome, particularly to understand the potential impact of pathogen exposure and the contribution of absent ASVs to the survival of captive-bred individuals following reintroduction.

An intriguing example that stands out for having a significantly lower relative abundance in captive samples is ASV 6, identified as *Arcicella* sp; commonly found in freshwater surface-dwelling microorganisms. Other examples of this disparity are ASV 29 and ASV 33, both *Rhodoferax* sp, a genus usually isolated from freshwater environments with putative detoxifying capacities [58]. On the other hand, certain bacteria are more relatively abundant in captive samples than wild ones, like ASV 7, a *Flavobacterium*. This genus is widespread in water-related places and can resist water-cleansing methods like chlorine [59] and antimicrobial products [60, 61]. Some *Flavobacterium* species possess antifungal properties [62], while others have been associated with increased relative abundance in physiologically stressed hosts [63], in some cases reaching levels that can be lethal for certain species [64]. Similarly, ASV 1 stands out as differentially relative abundant in W. Torreferrussa F0F1 (Fig. 2, Fig. 3, and Fig. S4), totally dominating this group’s microbiome. This ASV is an *Acinetobacter*, a functionally diverse genus that has been proven to have both *Bd-*inhibiting species [62] and pathogenic ones [65]. In the same way as *Flavobacterium*, *Acinetobacter* seems to thrive on amphibian skin when the host is under physiological stress [63], possibly highlighting a health issue in the W. Torreferrussa F0F1 population.

A concerning issue for future reintroduction efforts is the markedly lower microbiome diversity observed in captive-bred groups compared to their wild counterparts in both subspecies. This could be, among many reasons, due to a lack of bacterial diversity in their environment, resulting from the change from a natural environment to an artificial one, or the water treatment as part of each center’s policy. Although the extent of microbiome plasticity in *C. arnoldi* remains unknown, exposure to their natural habitat may be sufficient to restore a microbiome similar to that of wild individuals -or at least a different but functionally effective one. In amphibians, increases in skin microbiome diversity can occur after shedding even without environmental exposure, as bacteria recolonize the skin from elsewhere on the body surface; however, in the wild, this effect is likely amplified by immediate contact with diverse environmental microbes after shedding [66]. Captive conditions may therefore limit this natural amplification process, making it essential to provide the most realistic and ecologically relevant environment possible for captive-bred newts to facilitate successful reintroduction. This should include consideration of the natural composition and diversity of the *C. arnoldi* microbiome. The importance of maintaining a diverse microbiome lies in its role in reducing the risk of potential *Bd* and *Bsal* infections, as microbiome homogeneity has been associated with decreased survival when facing this disease [10], or other natural pathogens. Therefore, preserving or restoring microbiome diversity is a key factor in improving the reintroduction success of captive-bred *C. arnoldi* individuals into their natural environment.

One potential strategy to enhance microbiome diversity is the use of probiotic treatments, which have shown promising results in previous studies on other amphibians, like boreal toads (*Anaxyrus boreas*) [67]. In the long term, microbiome diversity could be enhanced within captive-breeding centers by gradually introducing natural substrates and water into the tanks -provided they are first screened for common amphibian pathogens. In the short term, and to avoid disrupting the current captive conditions, a soft-release strategy may be more effective. This approach involves temporarily confining individuals at the release site to allow acclimatization before full release [68], and has been shown to yield positive outcomes in some amphibian species [69].

This is particularly important when considering that amphibians can horizontally transmit skin bacteria [26]. In such cases, all individuals undergoing the soft-release procedure would eventually share the acquired bacteria upon reintroduction to the same location. Given the species’ microbiome plasticity, there is promise that reintroduced specimens can adapt their microbiome to withstand potential challenges better. Furthermore, the captive-bred *C. arnoldi* groups appeared to acquire different environmental bacteria compared to their wild counterparts and showed reduced relative abundance of several core ASVs that were consistently present in wild populations, which should be taken into account when considering probiotic treatments. In captive groups, these core ASVs were absent or present at much lower abundances, and the microbiomes were often dominated by other bacterial taxa. For instance, *Flectobacillus fontis* (ASV 18) was prevalent in captive-bred *C. a. arnoldi* groups, while *Flavobacterium* sp. (ASV 7) dominated the microbiome of captive-bred *C. a. laietanus* groups. Both taxa have also been detected in other captive amphibian species [26, 70], suggesting they may represent a microbial signature of captivity.

Moreover, some groups showed noticeable differences between the relative abundance of certain ASVs in their skin microbiome and in the surrounding water. For instance, individuals from the W. Pont de Suert F1 group exhibited high relative abundances of *Caenimonas* sp. on their skin, despite this ASV ranking 102nd out of 144 detected ASVs in their aquatic environment (Table S2). Specimens from the E. Barcelona Zoo F1F2 group also illustrated this mismatch, with ASV 25 being relatively abundant in their skin microbiomes despite ranking as the 145th most relatively abundant ASV in their surrounding water (Table S2). Although the idea that amphibians may selectively recruit rare environmental bacteria is well established [71], it remains intriguing that these individuals preferentially acquired such relatively low abundance ASVs over others like *Erwinia* sp. or *Arcinella* sp., which were more relatively abundant in the water and also commonly found in the wild populations’ skin microbiomes. Recent studies have demonstrated that amphibians actively select for specific bacterial taxa. For example, Loudon *et al*. [72] showed that amphibians preferentially select bacteria with antifungal properties that persist on the skin despite exposure to many environmental microbes. This indicates that, beyond environmental availability alone, host selection and interspecies microbial competition strongly shape skin bacterial communities, favoring taxa that provide functional benefits such as pathogen defense. However, the precise criteria determining which bacteria become established members of an individual’s microbiome remain poorly understood, but are likely influenced by a combination of host-specific traits, bacterial characteristics, host–microbe interactions, and environmental conditions. Given that amphibians may harbor bacterial communities particularly suited to their environmental and physiological needs and that these bacteria can be transmitted between individuals, further exploring phylosymbiosis in *C. arnoldi* would be of great interest. The concept of phylosymbiosis describes the coevolution of the species and their microbiomes in addition to environmental effects, tracing parallelisms between the microbial community and the host species. Therefore, it would mean that an amphibian microbial community can influence host evolution through composition and functional effects [73], which can in turn affect the species’ ecology, physiology, and behavior.

This study builds on previous work examining the microbiomes of wild and captive amphibians by providing the detailed characterization of the microbiome composition of *C. arnoldi*, one of Europe’s most threatened amphibians. Notably, the results also reveal *C. arnoldi*’s microbiome’s plasticity, as each captive-bred group developed significantly different microbiomes. While much remains to be understood, our findings raise important questions regarding reintroduction success. For instance, it is critical to assess whether microbiome composition is directly linked to survival after reintroduction, particularly in the face of pathogenic threats, or how the microbiome of released individuals will evolve. Will it reflect the community acquired in captivity, shift toward that of wild populations of the same subspecies, or develop into a distinct new assemblage? Moreover, establishing a baseline for the presence or absence of key bacterial taxa will be essential for designing future probiotic treatments, especially given the microbiome’s immunological role and the presence of *Bd* and *Bsal* in the region*.* In conclusion, a more comprehensive understanding of *C. arnoldi*’s microbiome and its ecological significance is vital for informing effective conservation strategies and ensuring the success of reintroduction programs.

## Supplementary Material

Fig_S1_ycaf245

Fig_S2_ycaf245

Fig_S3_ycaf245

Fig_S4_ycaf245

Table_S1_ycaf245

Table_S2_ycaf245

Table_S3_ycaf245

Table_S4_ycaf245

Table_S5_ycaf245

## Data Availability

The raw reads for the project have been deposited on NCBI SRA (BioProject: PRJNA1265703). Code used for sample processing is available in GitHub (https://github.com/delCampoLab/newt_microbiome). Raw read counts, taxonomy table, metadata, statistical results, relative abundances, core ASVs, and one-to-one ANCOM results are available with the Supplementary Material.
