# Supplementary material for: Comparative skin microbiome analyses reveal differences between wild populations and captive groups of the Montseny brook newt (Calotriton arnoldi)
Source: ISME Commun. 2026 Jan 8;6(1):ycaf245. doi: 10.1093/ismeco/ycaf245 (PMC12815265; doi:10.1093/ismeco/ycaf245)
Supplement: Fig_S1_ycaf245 [file fig_s1_ycaf245.pdf]

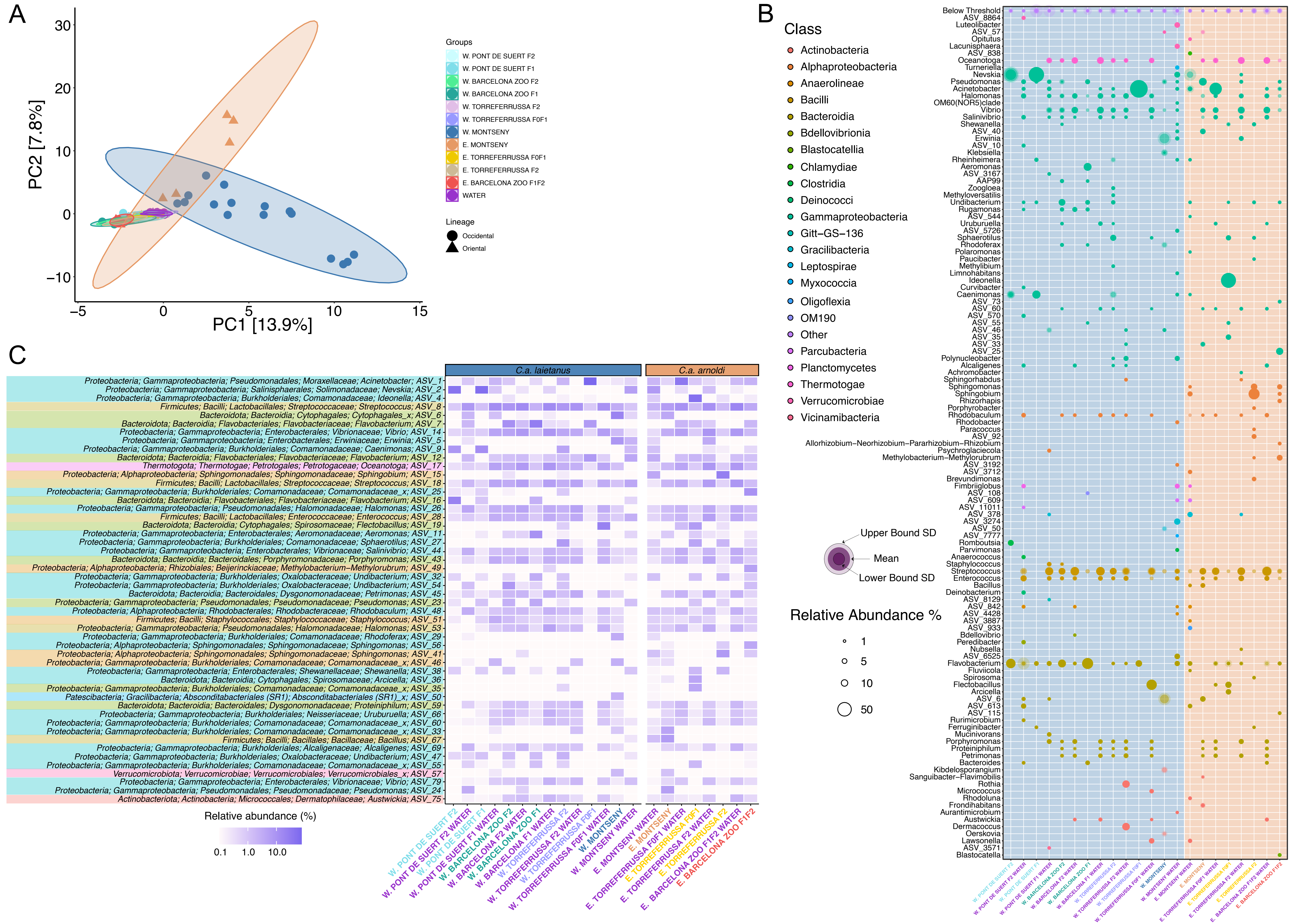

A
PC2 [7.8%]
PC1 [13.9%]
Groups
W. PONT DE SUERT F2
W. PONT DE SUERT F1
W. BARCELONA ZOO F2
W. BARCELONA ZOO F1
W. TORREFERRUSSA F2
W. TORREFERRUSSA F0F1
W. MONTSENY
E. MONTSENY
E. TORREFERRUSSA F0F1
E. TORREFERRUSSA F2
E. BARCELONA ZOO F1F2
WATER
Lineage
Occidental
Oriental
B
Class
Actinobacteria
Alphaproteobacteria
Anaerolineae
Bacilli
Bacteroidia
Bdellovibrionia
Blastocatellia
Chlamydiae
Clostridia
Deinococci
Gammaproteobacteria
Gitt–GS–136
Gracilibacteria
Leptospirae
Myxococcia
Oligoflexia
OM190
Other
Parcubacteria
Planctomycetes
Thermotogae
Verrucomicrobiae
Vicinamibacteria
Upper Bound SD
Mean
Lower Bound SD
Relative Abundance %
1
5
10
50
Below Threshold
ASV_8864
Luteolibacter
ASV_57
Opitutus
Lacunisphaera
ASV_838
Oceanotoga
Turneriella
Nevskia
Pseudomonas
Acinetobacter
Halomonas
OM60(NOR5)clade
Vibrio
Salinivibrio
Shewanella
ASV_40
Erwinia
ASV_10
Klebsiella
Rheinheimera
Aeromonas
ASV_3167
AAP99
Zoogloea
Methyloversatilis
Undibacterium
Rugamonas
ASV_544
Uruburuella
ASV_5726
Sphaerotilus
Rhodoferax
Polaromonas
Paucibacter
Methylibium
Limnohabitans
Ideonella
Curvibacter
Caenimonas
ASV_73
ASV_60
ASV_570
ASV_55
ASV_46
ASV_35
ASV_33
ASV_25
Polynucleobacter
Alcaligenes
Achromobacter
Sphingorhabdus
Sphingomonas
Sphingobium
Rhizorhapis
Porphyrobacter
Rhodobaculum
Rhodobacter
Paracoccus
ASV_92
Allorhizobium–Neorhizobium–Pararhizobium–Rhizobium
Psychroglaciecola
Methylobacterium–Methylorubrum
ASV_3192
ASV_3712
Brevundimonas
Fimbriiglobus
ASV_108
ASV_609
ASV_11011
ASV_378
ASV_3274
ASV_50
ASV_7777
Romboutsia
Parvimonas
Anaerococcus
Staphylococcus
Streptococcus
Enterococcus
Bacillus
Deinobacterium
ASV_8129
ASV_842
ASV_4428
ASV_3887
ASV_933
Bdellovibrio
Peredibacter
Nubsella
ASV_6525
Flavobacterium
Fluviicola
Spirosoma
Flectobacillus
Arcicella
ASV_6
ASV_613
ASV_115
Rurimicrobium
Ferruginibacter
Mucinivorans
Porphyromonas
Proteiniphilum
Petrimonas
Bacteroides
Kibdelosporangium
Sanguibacter–Flavimobilis
Rothia
Micrococcus
Rhodoluna
Frondihabitans
Aurantimicrobium
Austwickia
Dermacoccus
Oerskovia
Lawsonella
ASV_3571
Blastocatella
C
C.a. laietanus
C.a. arnoldi
Proteobacteria; Gammaproteobacteria; Pseudomonadales; Moraxellaceae; Acinetobacter; ASV_1
Proteobacteria; Gammaproteobacteria; Salinisphaerales; Solimonadaceae; Nevskia; ASV_2
Proteobacteria; Gammaproteobacteria; Burkholderiales; Comamonadaceae; Ideonella; ASV_4
Firmicutes; Bacilli; Lactobacillales; Streptococcaceae; Streptococcus; ASV_8
Bacteroidota; Bacteroidia; Cytophagales; Cytophagales_x; ASV_6
Bacteroidota; Bacteroidia; Flavobacteriales; Flavobacteriaceae; Flavobacterium; ASV_7
Proteobacteria; Gammaproteobacteria; Enterobacterales; Vibrionaceae; Vibrio; ASV_14
Proteobacteria; Gammaproteobacteria; Enterobacterales; Erwiniaceae; Erwinia; ASV_5
Proteobacteria; Gammaproteobacteria; Burkholderiales; Comamonadaceae; Caenimonas; ASV_9
Bacteroidota; Bacteroidia; Flavobacteriales; Flavobacteriaceae; Flavobacterium; ASV_12
Thermotogota; Thermotogae; Petrotogales; Petrotogaceae; Oceanotoga; ASV_17
Proteobacteria; Alphaproteobacteria; Sphingomonadales; Sphingomonadaceae; Sphingobium; ASV_15
Firmicutes; Bacilli; Lactobacillales; Streptococcaceae; Streptococcus; ASV_18
Proteobacteria; Gammaproteobacteria; Burkholderiales; Comamonadaceae; Comamonadaceae_x; ASV_25
Bacteroidota; Bacteroidia; Flavobacteriales; Flavobacteriaceae; Flavobacterium; ASV_16
Proteobacteria; Gammaproteobacteria; Pseudomonadales; Halomonadaceae; Halomonas; ASV_26
Firmicutes; Bacilli; Lactobacillales; Enterococcaceae; Enterococcus; ASV_28
Bacteroidota; Bacteroidia; Cytophagales; Spirosomaceae; Flectobacillus; ASV_19
Proteobacteria; Gammaproteobacteria; Enterobacterales; Aeromonadaceae; Aeromonas; ASV_11
Proteobacteria; Gammaproteobacteria; Burkholderiales; Comamonadaceae; Sphaerotilus; ASV_27
Proteobacteria; Gammaproteobacteria; Enterobacterales; Vibrionaceae; Salinivibrio; ASV_44
Bacteroidota; Bacteroidia; Bacteroidales; Porphyromonadaceae; Porphyromonas; ASV_43
Proteobacteria; Alphaproteobacteria; Rhizobiales; Beijerinckiaceae; Methylobacterium–Methylorubrum; ASV_49
Proteobacteria; Gammaproteobacteria; Burkholderiales; Oxalobacteraceae; Undibacterium; ASV_32
Proteobacteria; Gammaproteobacteria; Burkholderiales; Oxalobacteraceae; Undibacterium; ASV_54
Bacteroidota; Bacteroidia; Bacteroidales; Dysgonomonadaceae; Petrimonas; ASV_45
Proteobacteria; Gammaproteobacteria; Pseudomonadales; Pseudomonadaceae; Pseudomonas; ASV_23
Proteobacteria; Alphaproteobacteria; Rhodobacterales; Rhodobacteraceae; Rhodobaculum; ASV_48
Firmicutes; Bacilli; Staphylococcales; Staphylococcaceae; Staphylococcus; ASV_51
Proteobacteria; Gammaproteobacteria; Pseudomonadales; Halomonadaceae; Halomonas; ASV_53
Proteobacteria; Gammaproteobacteria; Burkholderiales; Comamonadaceae; Rhodoferax; ASV_29
Proteobacteria; Alphaproteobacteria; Sphingomonadales; Sphingomonadaceae; Sphingomonas; ASV_56
Proteobacteria; Alphaproteobacteria; Sphingomonadales; Sphingomonadaceae; Sphingomonas; ASV_41
Proteobacteria; Gammaproteobacteria; Burkholderiales; Comamonadaceae; Comamonadaceae_x; ASV_46
Proteobacteria; Gammaproteobacteria; Enterobacterales; Shewanellaceae; Shewanella; ASV_38
Bacteroidota; Bacteroidia; Cytophagales; Spirosomaceae; Arcicella; ASV_36
Proteobacteria; Gammaproteobacteria; Burkholderiales; Comamonadaceae; Comamonadaceae_x; ASV_35
Patescibacteria; Gracilibacteria; Absconditabacteriales (SR1); Absconditabacteriales (SR1)_x; ASV_50
Bacteroidota; Bacteroidia; Bacteroidales; Dysgonomonadaceae; Proteiniphilum; ASV_59
Proteobacteria; Gammaproteobacteria; Burkholderiales; Neisseriaceae; Uruburuella; ASV_66
Proteobacteria; Gammaproteobacteria; Burkholderiales; Comamonadaceae; Comamonadaceae_x; ASV_60
Proteobacteria; Gammaproteobacteria; Burkholderiales; Comamonadaceae; Comamonadaceae_x; ASV_33
Firmicutes; Bacilli; Bacillales; Bacillaceae; Bacillus; ASV_67
Proteobacteria; Gammaproteobacteria; Burkholderiales; Alcaligenaceae; Alcaligenes; ASV_69
Proteobacteria; Gammaproteobacteria; Burkholderiales; Oxalobacteraceae; Undibacterium; ASV_47
Proteobacteria; Gammaproteobacteria; Burkholderiales; Comamonadaceae; Comamonadaceae_x; ASV_55
Verrucomicrobiota; Verrucomicrobiae; Verrucomicrobiales; Verrucomicrobiales_x; ASV_57
Proteobacteria; Gammaproteobacteria; Enterobacterales; Vibrionaceae; Vibrio; ASV_79
Proteobacteria; Gammaproteobacteria; Pseudomonadales; Pseudomonadaceae; Pseudomonas; ASV_24
Actinobacteriota; Actinobacteria; Micrococcales; Dermatophilaceae; Austwickia; ASV_75
Relative abundance (%)
0.1
1.0
10.0
W. PONT DE SUERT F2
W. PONT DE SUERT F2 WATER
W. PONT DE SUERT F1
W. PONT DE SUERT F1 WATER
W. BARCELONA ZOO F2
W. BARCELONA ZOO F2 WATER
W. BARCELONA ZOO F1
W. BARCELONA F1 WATER
W. TORREFERRUSSA F2
W. TORREFERRUSSA F2 WATER
W. TORREFERRUSSA F0F1
W. TORREFERRUSSA F0F1 WATER
W. MONTSENY
W. MONTSENY WATER
E. MONTSENY WATER
E. MONTSENY
E. TORREFERRUSSA F0F1 WATER
E. TORREFERRUSSA F0F1
E. TORREFERRUSSA F2 WATER
E. TORREFERRUSSA F2
E. BARCELONA ZOO F1F2 WATER
E. BARCELONA ZOO F1F2
