# Supplementary material for: Comparative skin microbiome analyses reveal differences between wild populations and captive groups of the Montseny brook newt (Calotriton arnoldi)
Source: ISME Commun. 2026 Jan 8;6(1):ycaf245. doi: 10.1093/ismeco/ycaf245 (PMC12815265; doi:10.1093/ismeco/ycaf245)
Supplement: Fig_S2_ycaf245 [file fig_s2_ycaf245.pdf]

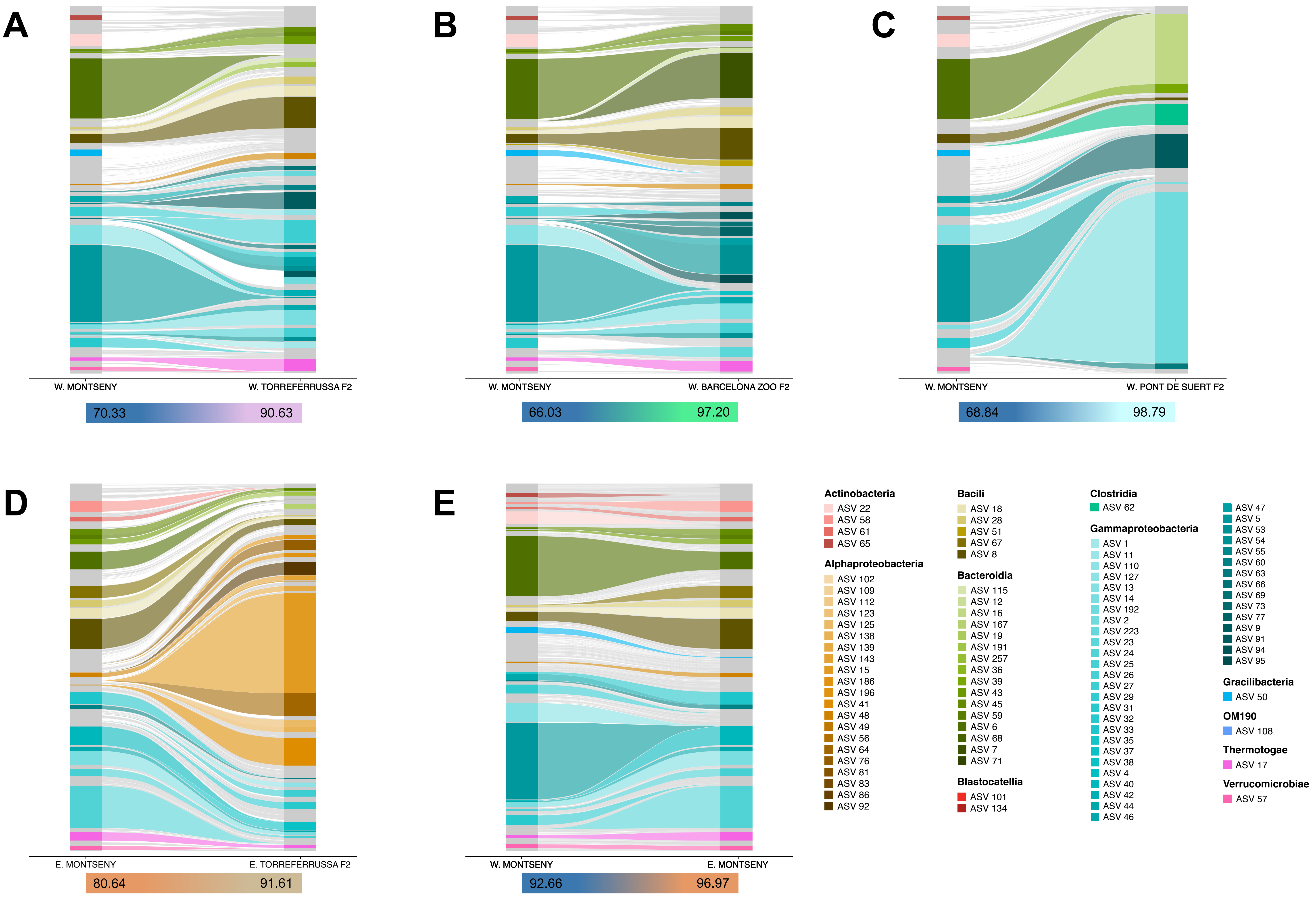
A
W. MONTSENY
W. TORREFERRUSSA F2
70.33
90.63
B
W. MONTSENY
W. BARCELONA ZOO F2
66.03
97.20
C
W. MONTSENY
W. PONT DE SUERT F2
68.84
98.79
D
E. MONTSENY
E. TORREFERRUSSA F2
80.64
91.61
E
W. MONTSENY
E. MONTSENY
92.66
96.97
Actinobacteria
ASV 22
ASV 58
ASV 61
ASV 65
Alphaproteobacteria
ASV 102
ASV 109
ASV 112
ASV 123
ASV 125
ASV 138
ASV 139
ASV 143
ASV 15
ASV 186
ASV 196
ASV 41
ASV 48
ASV 49
ASV 56
ASV 64
ASV 76
ASV 81
ASV 83
ASV 86
ASV 92
Bacili
ASV 18
ASV 28
ASV 51
ASV 67
ASV 8
Bacteroidia
ASV 115
ASV 12
ASV 16
ASV 167
ASV 19
ASV 191
ASV 257
ASV 36
ASV 39
ASV 43
ASV 45
ASV 59
ASV 6
ASV 68
ASV 7
ASV 71
Blastocatellia
ASV 101
ASV 134
Clostridia
ASV 62
Gammaproteobacteria
ASV 1
ASV 11
ASV 110
ASV 127
ASV 13
ASV 14
ASV 192
ASV 2
ASV 223
ASV 23
ASV 24
ASV 25
ASV 26
ASV 27
ASV 29
ASV 31
ASV 32
ASV 33
ASV 35
ASV 37
ASV 38
ASV 4
ASV 40
ASV 42
ASV 44
ASV 46
ASV 47
ASV 5
ASV 53
ASV 54
ASV 55
ASV 60
ASV 63
ASV 66
ASV 69
ASV 73
ASV 77
ASV 9
ASV 91
ASV 94
ASV 95
Gracilibacteria
ASV 50
OM190
ASV 108
Thermotogae
ASV 17
Verrucomicrobiae
ASV 57
