# Supplementary material for: Comparative skin microbiome analyses reveal differences between wild populations and captive groups of the Montseny brook newt (Calotriton arnoldi)
Source: ISME Commun. 2026 Jan 8;6(1):ycaf245. doi: 10.1093/ismeco/ycaf245 (PMC12815265; doi:10.1093/ismeco/ycaf245)
Supplement: Fig_S3_ycaf245 [file fig_s3_ycaf245.pdf]

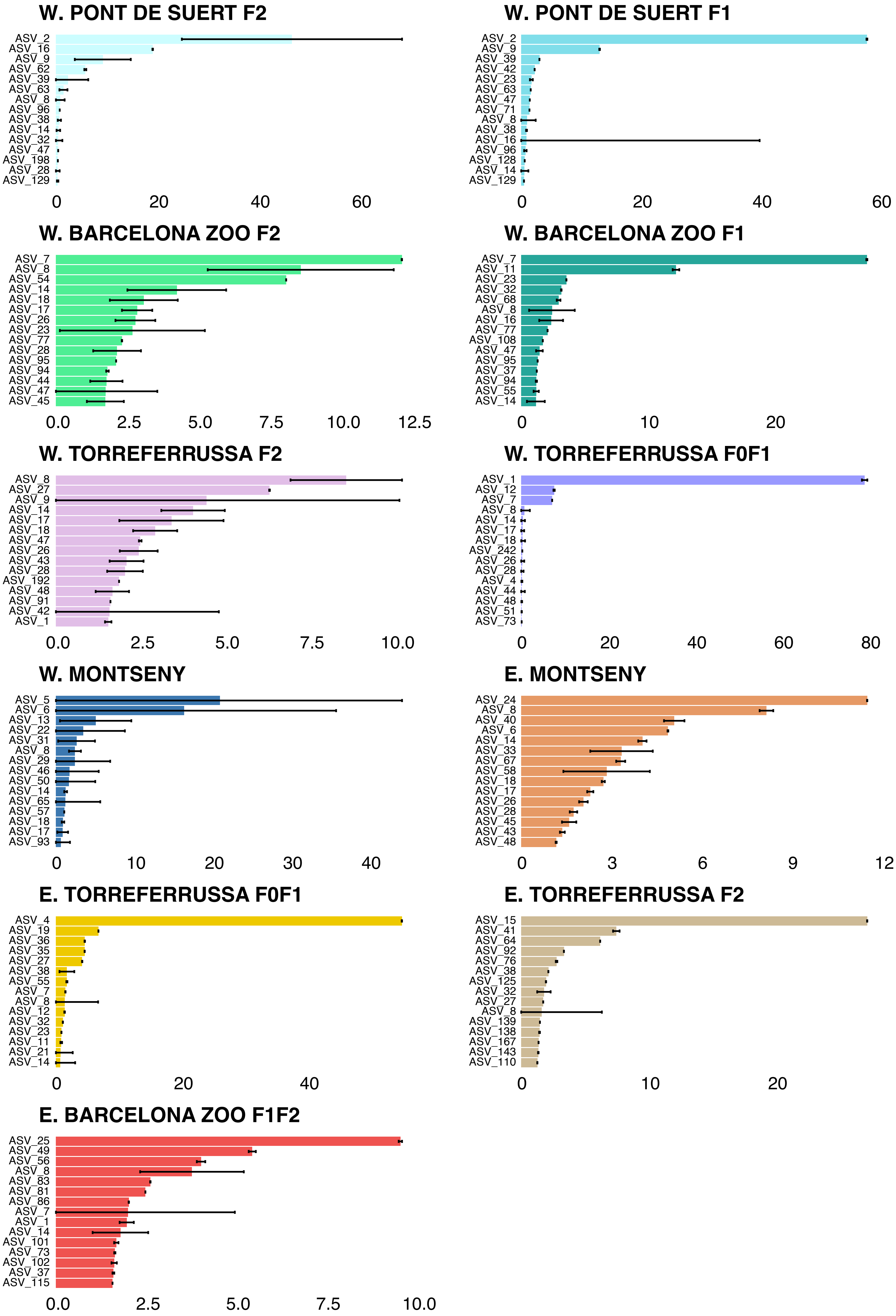

W. PONT DE SUERT F2
ASV_2
ASV_16
ASV_9
ASV_62
ASV_39
ASV_63
ASV_8
ASV_96
ASV_38
ASV_14
ASV_32
ASV_47
ASV_198
ASV_28
ASV_129
0
20
40
60
W. PONT DE SUERT F1
ASV_2
ASV_9
ASV_39
ASV_42
ASV_23
ASV_63
ASV_47
ASV_71
ASV_8
ASV_38
ASV_16
ASV_96
ASV_128
ASV_14
ASV_129
0
20
40
60
W. BARCELONA ZOO F2
ASV_7
ASV_8
ASV_54
ASV_14
ASV_18
ASV_17
ASV_26
ASV_23
ASV_77
ASV_28
ASV_95
ASV_94
ASV_44
ASV_47
ASV_45
0.0
2.5
5.0
7.5
10.0
12.5
W. BARCELONA ZOO F1
ASV_7
ASV_11
ASV_23
ASV_32
ASV_68
ASV_8
ASV_16
ASV_77
ASV_108
ASV_47
ASV_95
ASV_37
ASV_94
ASV_55
ASV_14
0
10
20
W. TORREFERRUSSA F2
ASV_8
ASV_27
ASV_9
ASV_14
ASV_17
ASV_18
ASV_47
ASV_26
ASV_43
ASV_28
ASV_192
ASV_48
ASV_91
ASV_42
ASV_1
0.0
2.5
5.0
7.5
10.0
W. TORREFERRUSSA F0F1
ASV_1
ASV_12
ASV_7
ASV_8
ASV_14
ASV_17
ASV_18
ASV_242
ASV_26
ASV_28
ASV_4
ASV_44
ASV_48
ASV_51
ASV_73
0
20
40
60
80
W. MONTSENY
ASV_5
ASV_6
ASV_13
ASV_22
ASV_31
ASV_8
ASV_29
ASV_46
ASV_50
ASV_14
ASV_65
ASV_57
ASV_18
ASV_17
ASV_93
0
10
20
30
40
E. MONTSENY
ASV_24
ASV_8
ASV_40
ASV_6
ASV_14
ASV_33
ASV_67
ASV_58
ASV_18
ASV_17
ASV_26
ASV_28
ASV_45
ASV_43
ASV_48
0
3
6
9
12
E. TORREFERRUSSA F0F1
ASV_4
ASV_19
ASV_36
ASV_35
ASV_27
ASV_38
ASV_55
ASV_7
ASV_8
ASV_12
ASV_32
ASV_23
ASV_11
ASV_21
ASV_14
0
20
40
E. TORREFERRUSSA F2
ASV_15
ASV_41
ASV_64
ASV_92
ASV_76
ASV_38
ASV_125
ASV_32
ASV_27
ASV_8
ASV_139
ASV_138
ASV_167
ASV_143
ASV_110
0
10
20
E. BARCELONA ZOO F1F2
ASV_25
ASV_49
ASV_56
ASV_8
ASV_83
ASV_81
ASV_86
ASV_7
ASV_1
ASV_14
ASV_101
ASV_73
ASV_102
ASV_37
ASV_115
0.0
2.5
5.0
7.5
10.0
