# Supplementary figures and images for: Comparative skin microbiome analyses reveal differences between wild populations and captive groups of the Montseny brook newt (Calotriton arnoldi)
Source: ISME Commun. 2026 Jan 8;6(1):ycaf245. doi: 10.1093/ismeco/ycaf245 (PMC12815265; doi:10.1093/ismeco/ycaf245)

-log10 (adjusted p-value)

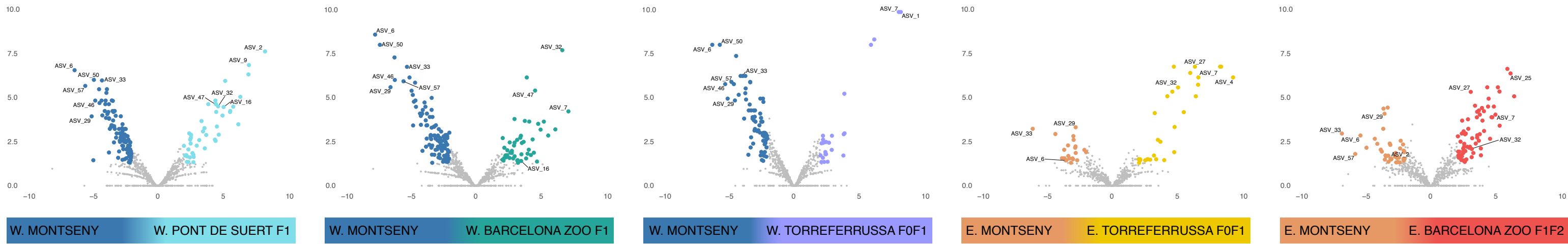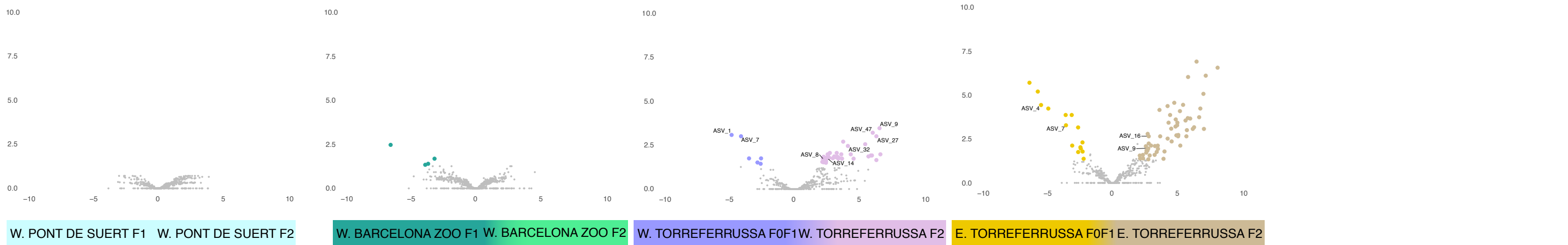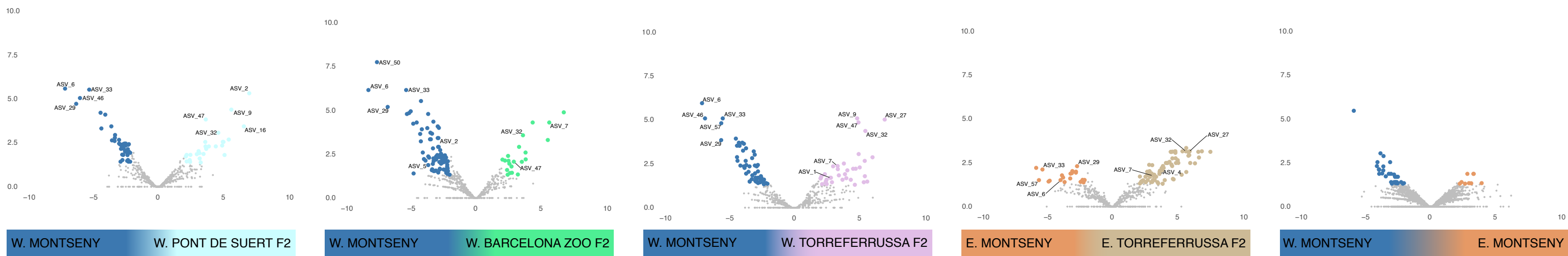

Log-fold Change

Supplement: Fig_S4_ycaf245 [file fig_s4_ycaf245.pdf]
